# Reproducibility of structural brain connectivity and network metrics using probabilistic diffusion tractography

**DOI:** 10.1038/s41598-018-29943-0

**Published:** 2018-08-01

**Authors:** Shang-Yueh Tsai

**Affiliations:** 1 Graduate Institute of Applied Physics, National Chengchi University, Taipei, Taiwan; 2 Research Center for Mind, Brain and Learning, National Chengchi University, Taipei, Taiwan

## Abstract

The structural connectivity network constructed using probabilistic diffusion tractography can be characterized by the network metrics. In this study, short-term test-retest reproducibility of structural networks and network metrics were evaluated on 30 subjects in terms of within- and between-subject coefficient of variance (CV_ws_, CV_bs_), and intra class coefficient (ICC) using various connectivity thresholds. The short-term reproducibility under various connectivity thresholds were also investigated when subject groups have same or different sparsity. In summary, connectivity threshold of 0.01 can exclude around 80% of the edges with CV_ws_ = 73.2 ± 37.7%, CV_bs_ = 119.3 ± 44.0% and ICC = 0.62 ± 0.19. The rest 20% edges have CV_ws_ < 45%, CV_bs_ < 90%, ICC = 0.75 ± 0.12. The presence of 1% difference in the sparsity can cause additional within-subject variations on network metrics. In conclusion, applying connectivity thresholds on structural network to exclude spurious connections for the network analysis should be considered as necessities. Our findings suggest that a connectivity threshold over 0.01 can be applied without significant effect on the short-term when network metrics are evaluated at the same sparsity in subject group. When the sparsity is not the same, the procedure of integration over various connectivity thresholds can provide reliable estimation of network metrics.

## Introduction

Diffusion Tensor Imaging (DTI) and the associate tractography methods can be used to reconstruct fiber bundles based on voxel based directional information^1–6^. Based on the tractography, the connectivity matrix also known as structural network can be constructed from structural connectivity calculated by counting the number of fibers between cortical regions in the brain^7–10^. Further, graph theory-based network analysis has been applied on the connectivity matrix to investigate the topology properties of entire network instead of individual analysis of large number of tracts^11–14^. The network metrics such as global efficiency, cluster coefficients have been found to be relevant in age and gender of healthy human brain^15,16^. The change in network metrics have already been found in several neurological and psychological disorders^17–21^.

The network metrics can be estimated in various ways with several intermediate steps. The reproducibility of network metrics can be therefore affected by many factors. For example: the number of directions of DTI acquisition, structural connectivity estimated from deterministic or probabilistic tractogaphy, the structural network in binary or weighted, the definition of weighting of weighted network, the number of nodes in the structural network, different kind of thresholds applied on the connectivity. Because potential sources of bias and errors in each step can induce the variability of the outcome measures, a full characterization of the reproducibility of structural network and of network metrics is therefore essential for the applications^22–25^. In summary, Vaessen and associates^25^ reported the within subject coefficient of variation (CV) of network metrics for three kinds of DTI scheme (6, 15, 32 directions) for binary structural network. Owen and colleagues^24^ reported the CV and intraclass correlation coefficient (ICC) for intra- and inter-site to address the issue on the number of nodes for both binary and weighted network. Buchanan and colleagues^26^ compared the repeatability of network metrics from weighted network using two tractography algorithms (deterministic and probabilistic), two seeding approaches (white matter and gray matter), and three definitions of network weightings. They showed that probabilistic tractography has higher ICC on network metrics. Bonilha and colleagues^27^ also showed that probabilistic tractography has higher ICC on network metrics. Andreotti and colleagues^23^ reported the CV and ICC for global and local network metrics and addressed the issues on the effect of density thresholds. In their report, the weighted network is constructed using a connectivity threshold to exclude edges having low connectivity and using a density threshold to maintain the total number of edges (sparsity) across subjects, which is considered important in the comparison of network metrics between subjects^28,29^. They showed that applying the density and connectivity thresholds has significant effect on the network metrics and improves the reliability of network metrics (ICC and CV). Although there is argument that connectivity thresholds should not be applied or it will eliminate the real network property of individuals^24,26^, the strategy of connectivity thresholds have been adopted in studying age and gender differences of network metrics^15^, and in studying patients with attention-deficit/hyperactivity disorder^21^. In these studies, connectivity thresholds were applied on whole subject groups to exclude edges. In this way, the structural network has same sparsity and identical position of the connections for all subjects.

The probabilistic approach is considered more effective in the calculation of connectivity between cortical regions compared to the deterministic approach^27,30,31^. However, little is known about the reproducibility of the connectivity matrix constructed from probabilistic tractography and how the connectivity thresholds affect the reproducibility of network metrics. We think the issues about what connectivity thresholds should be applied can be addressed by investigating the reproducibility of structural connectivity at different connectivity levels. Then, the reproducibility of network metrics at various connectivity thresholds can be reported. Because the sparsity may differ when connectivity thresholds are applied, it is important understand how the reproducibility of the network metrics alter when the sparsity is not the same. In addition, the strategy of cost normalization is used in the calculation of network metrics to maintain the sum of all weighting at the same level^15,16,21^. It is interested to investigate the reproducibility of the network metrics before and after cost normalization.

In this study, we constructed the structural connectivity matrix among cortical regions using probabilistic tractography from repeated scans on healthy subjects. We calculated the network metrics based on weighted structural network. Three specific issues are investigated. (1) We investigate the short-term reproducibility of structural connectivity matrix at various levels of connectivity. (2) We investigate the short-term reproducibility of network metrics at various connectivity thresholds. (3) We investigate the effects of three processing strategies on the short-term reproducibility of network metrics including integration over all connectivity thresholds, maintenance of sparsity and cost normalization.

## Results

### Structural connectivity matrix

**Figure 1:**
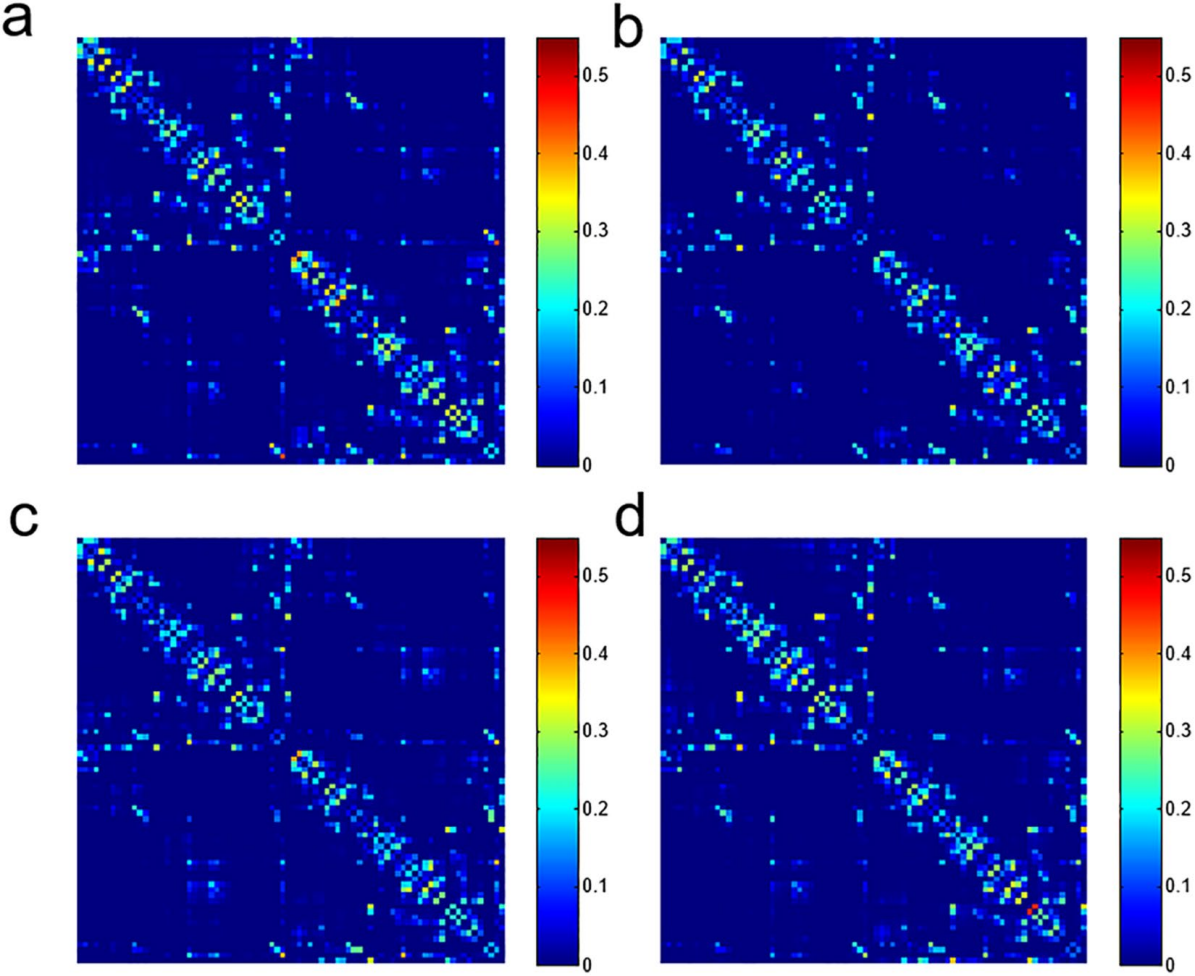
Structural connectivity matrices of four subjects. Each matrix element (edge) represents the weighted connectivity (0 to 1) from probabilistic tractography between cortical regions by AAL template. The 78 cortical regions are in left to right hemisphere order and names of cortical regions are listed in Supplementary Table 1.

**Figure 2:**
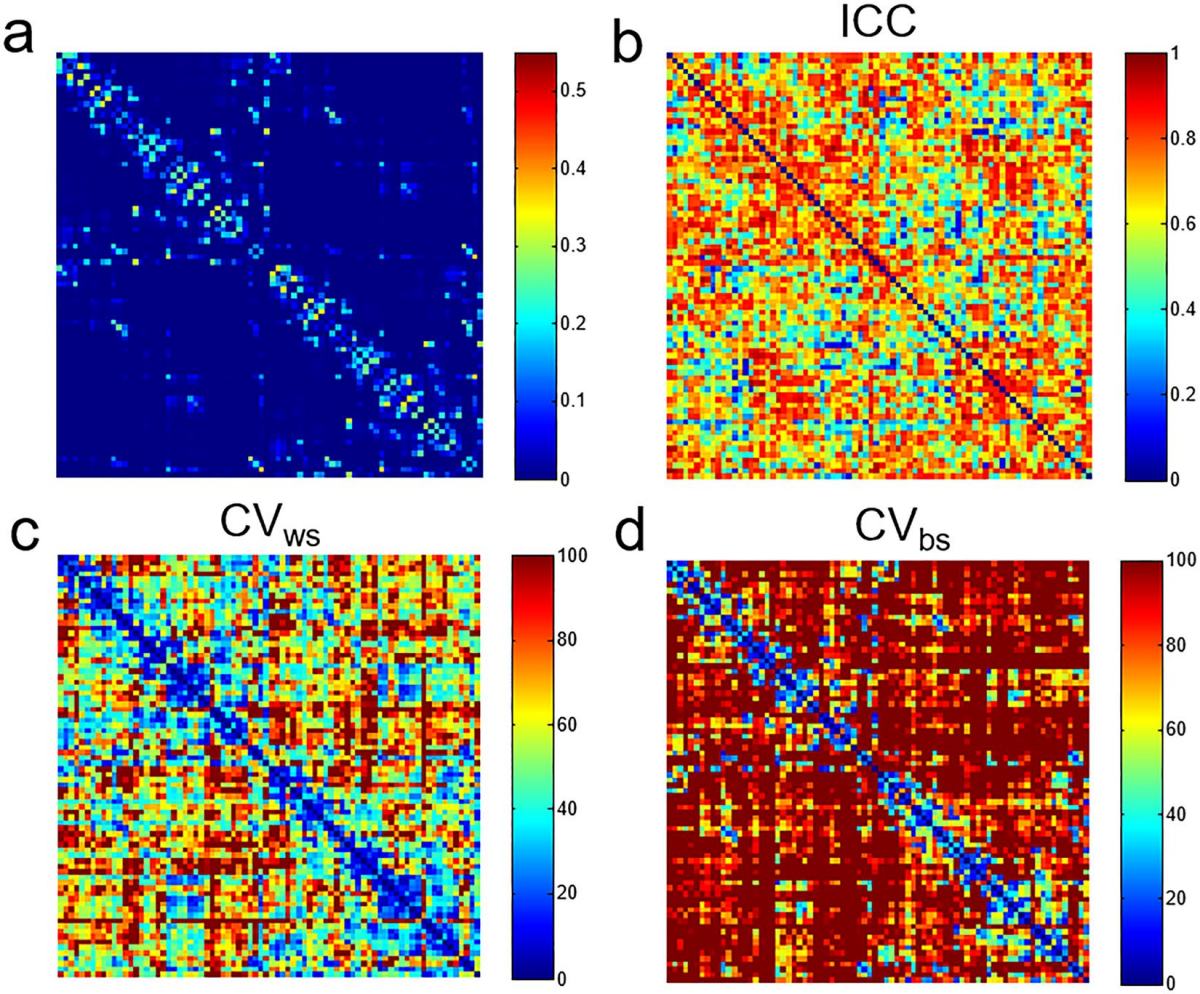
(**a**) Averaged connectivity matrix from 30 subjects and matrices of (**b**) ICC, (**c**) CV_ws_ (%), (**d**) CV_bs_ (%). Note edges with higher connectivity have lower CV_ws_ and CV_bs_.

**Figure 3:**
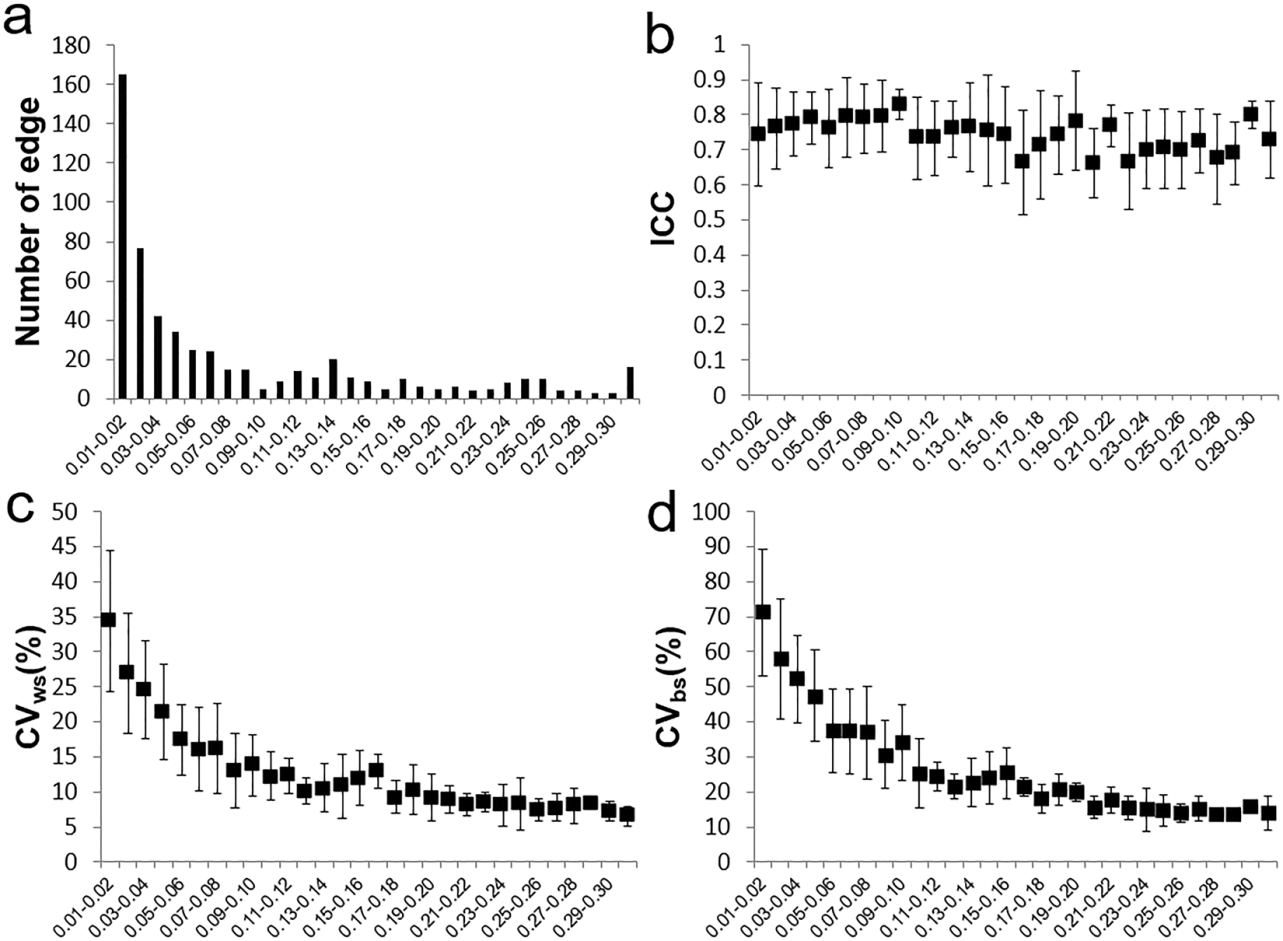
(**a**) Histogram of number of edges versus connectivity groups (0.01 to 0.30 with 0.01 steps) and corresponding (**b**) ICC, (**c**) CV_ws_ (%), (**d**) CV_bs_ (%) of 30 groups. For connectivity less than 0.01, there are 2428 (80.85%) out of total 3003 edges with the CV_bs_ (118 ± 44.5%), CV_ws_ (73.2 ± 37.7%) and ICC (0.62 ± 0.19).

Figure 1 shows the structural connectivity matrixes of 78 cortical regions from 4 subjects. Edges with stronger connectivity are mostly along the diagonal line and aside in the left-upper and right-lower part of the matrix. These edges belong to link with shorter inter-region distance and ROI sizes are larger in these regions. The within subject similarity of the connectivity of all subjects are 0.98 for and between subject similarity is 0.95. The distribution of structural connectivity matrix is similar to those in the previous reports using probabilistic tractography^15,21,27^. The averaged structural connectivity for 30 subjects with corresponding matrix of CV_bs_, CV_ws_ and ICC are shown in Fig. 2. The edges with higher connectivity have lower CV_bs_ and CV_ws_. Further, CV_ws_ are in general lower than CV_bs_. The histogram of the number of edges versus connectivity (connectivity >0.01) and corresponding CV_bs_, CV_ws_ and ICC are shown in Fig. 3. Overall, both CV_bs_ and CV_ws_ decrease as increasing connectivity. The CV_bs_ are all higher than CV_ws_. The ICC of the edges with connectivity over 0.01 is 0.75 ± 0.12 and the mean of ICC in each group range from 0.66 to 0.83. No observable trend in ICC versus connectivity is found but the variations of CV_ws_ and CV_bs_ decrease for edges with connectivity over 0.1. For all 3003 edges, there are 2428 (80.85%) of the edges having connectivity less than 0.01. A significant increase of the CV_bs_ (118 ± 44.5%) and CV_ws_ (73.2 ± 37.7%) is found for edges with connectivity less than 0.01 and the ICC of this group is 0.62 ± 0.19.

### Network metrics

**Figure 4:**
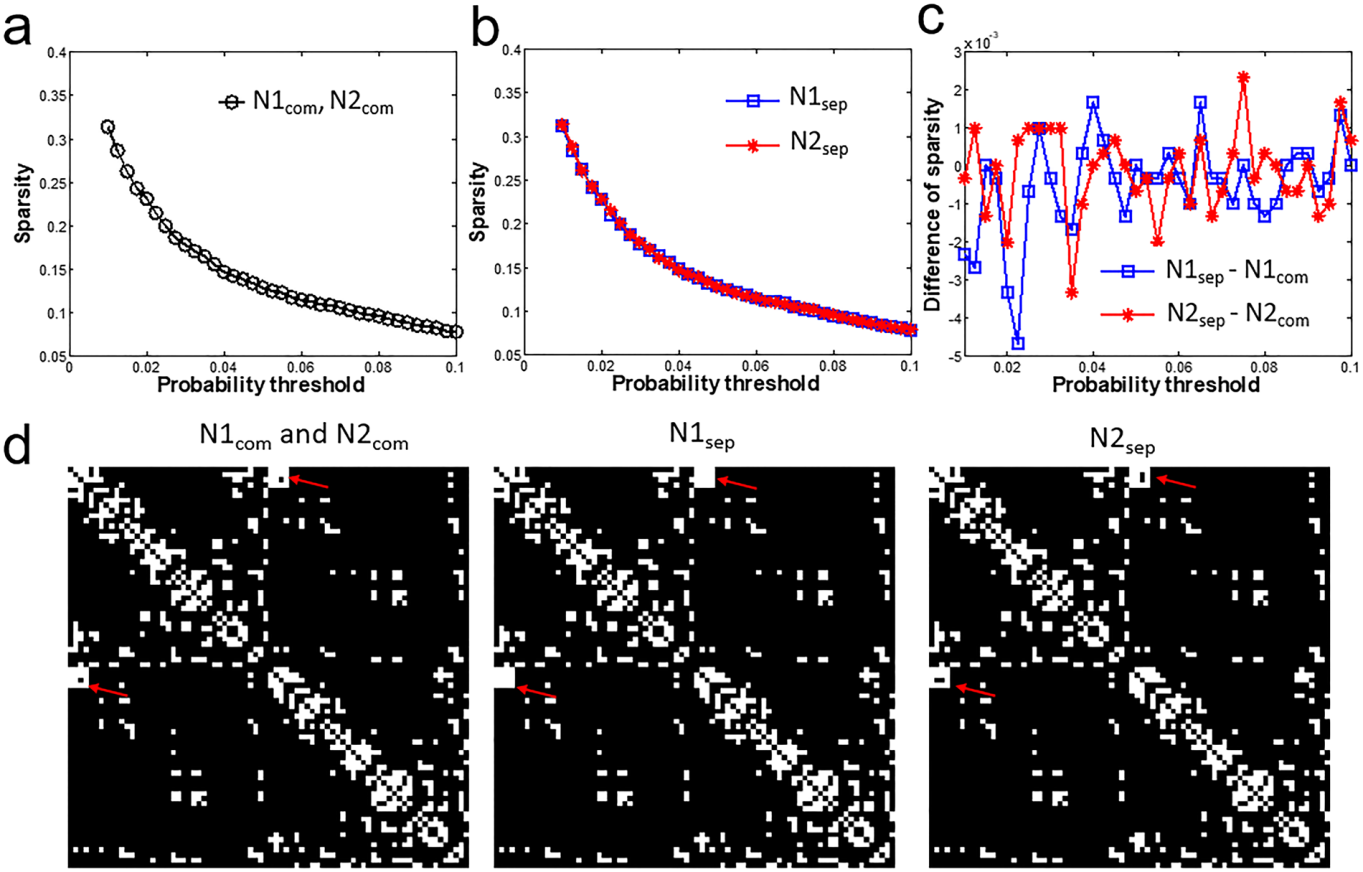
The sparsity at 37 connectivity thresholds from 0.01 to 0.10 with a step of 0.0025. The connectivity thresholds are applied on (**a**) N1 and N2 together (N1_com_ and N2_com_) and (**b**) N1 and N2 separately (N1_sep_ and N2_sep_). (**c**) Difference of N1_sep_ to N1_com_ and of N2_sep_ and N2_com_. Note the sparsity decrease from 0.32 to 0.08 as increasing connectivity thresholds and the difference in sparsity is less than 5 × 10^−3^. (**d**) masks of connectivity matrix at connectivity thresholds of 0.0575 where the sparsity is 0.1171 for N1_com_, N2_com_, 0.1182 for N1_sep_, and 0.1175 for N2_sep_. The differences on the edges are indicated by the red arrow.

The sparsity for connectivity thresholds applied together and separate are shown in Fig. 4. The sparsity decrease from 0.32 to 0.08 as connectivity thresholds increasing from 0.01 to 0.1. The sparsity is the same for N1_com_ and N2_com_. A slight difference is found in the sparsity between N1_sep_ and N2_sep_ (Fig. 4b). The sparsity of N1_sep_ and N2_sep_ also differ from N1_com_ and N2_com_ in the range of −4.7 × 10^−3^ to 2.3 × 10^−3^ (Fig. 4c). Whether the thresholds are applied together and separate, the spatial distribution of edges on the connectivity matrix is similar at the same threshold (Fig. 4d).

**Figure 5:**
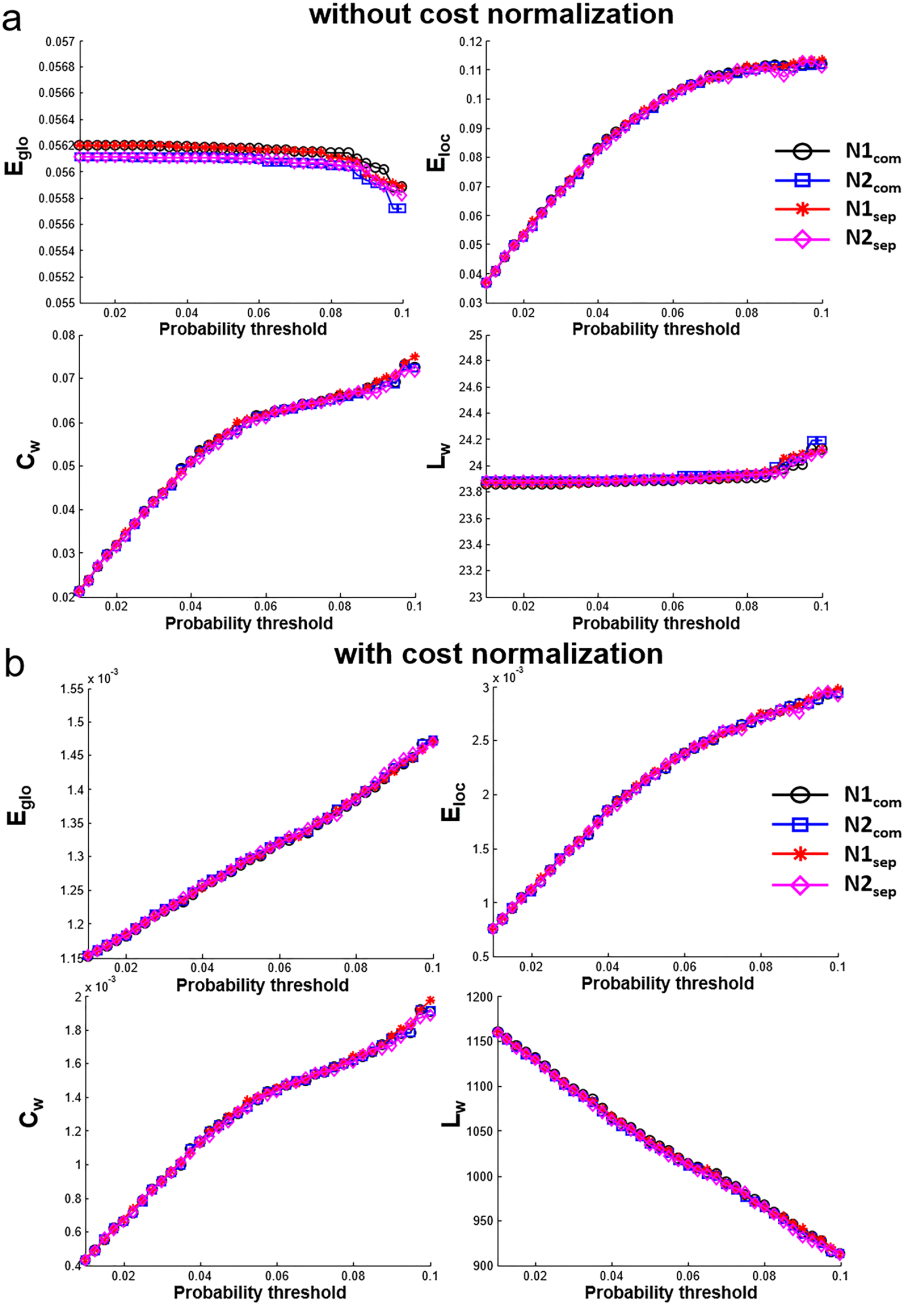
Network metrics (E_glo_, E_loc_, C_w_, L_w_) of N1_com_, N2_com_, N1_sep_, N2_sep_ at 37 connectivity thresholds estimated (**a**) without cost normalization and (**b**) with cost normalization. Note that all network metrics have consistent trend o as increasing connectivity thresholds and minor difference can be observed on the network metrics among N1_com_, N2_com_, N1_sep_, N2_sep_.

**Figure 6:**
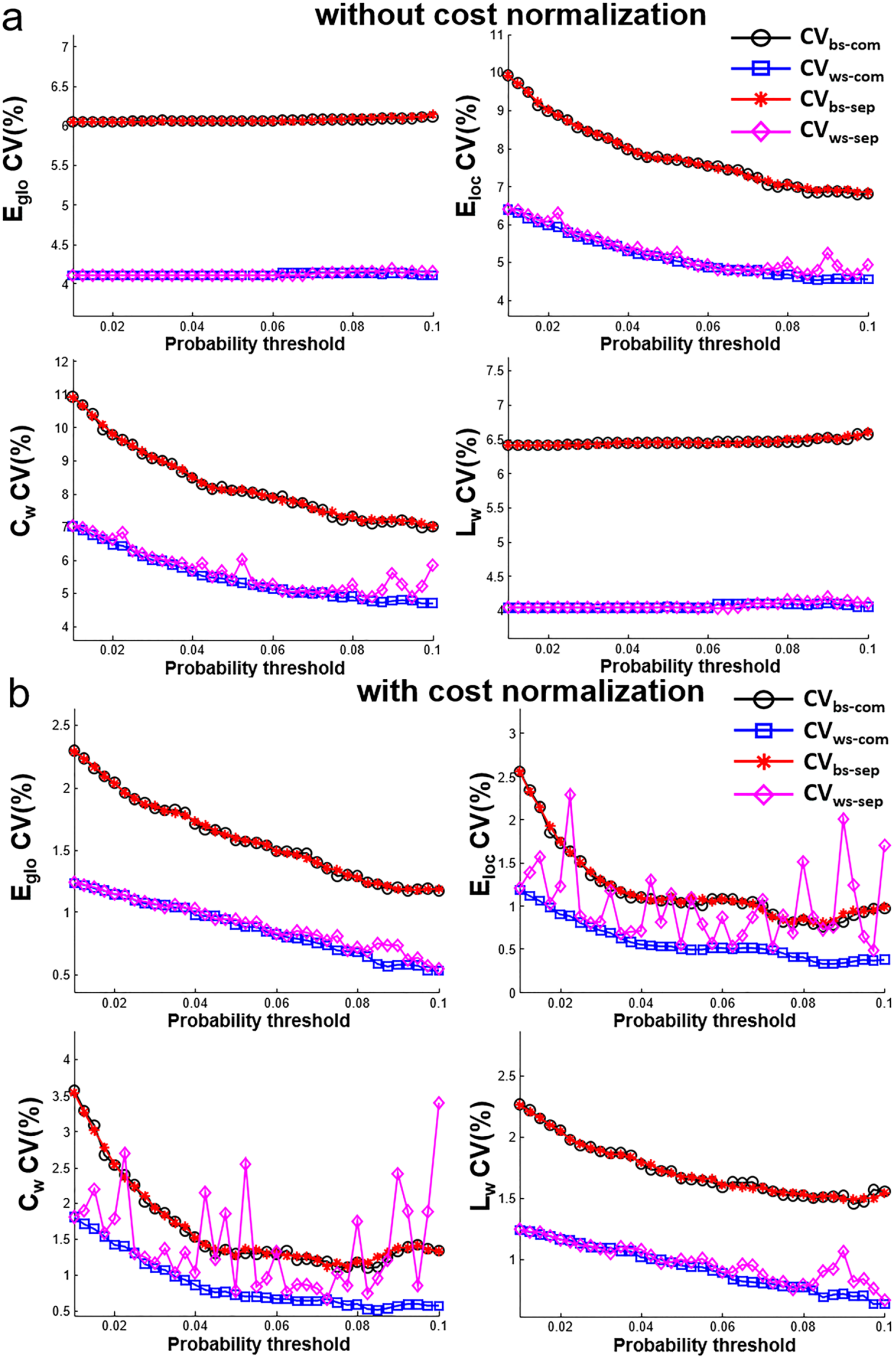
CV_ws_ and CV_bs_ of network metrics (E_glo_, E_loc_, C_w_, L_w_) at 37 connectivity thresholds estimated (**a**) without cost normalization and (**b**) with cost normalization. Both CV_bs_ and CV_ws_ vary smoothly as increasing connectivity thresholds. When sparsity is not the same (N1_sep_ and N2_sep_), dramatic variations of CV_ws_ are found in E_loc_, C_w_, estimated with cost normalization.

**Figure 7:**
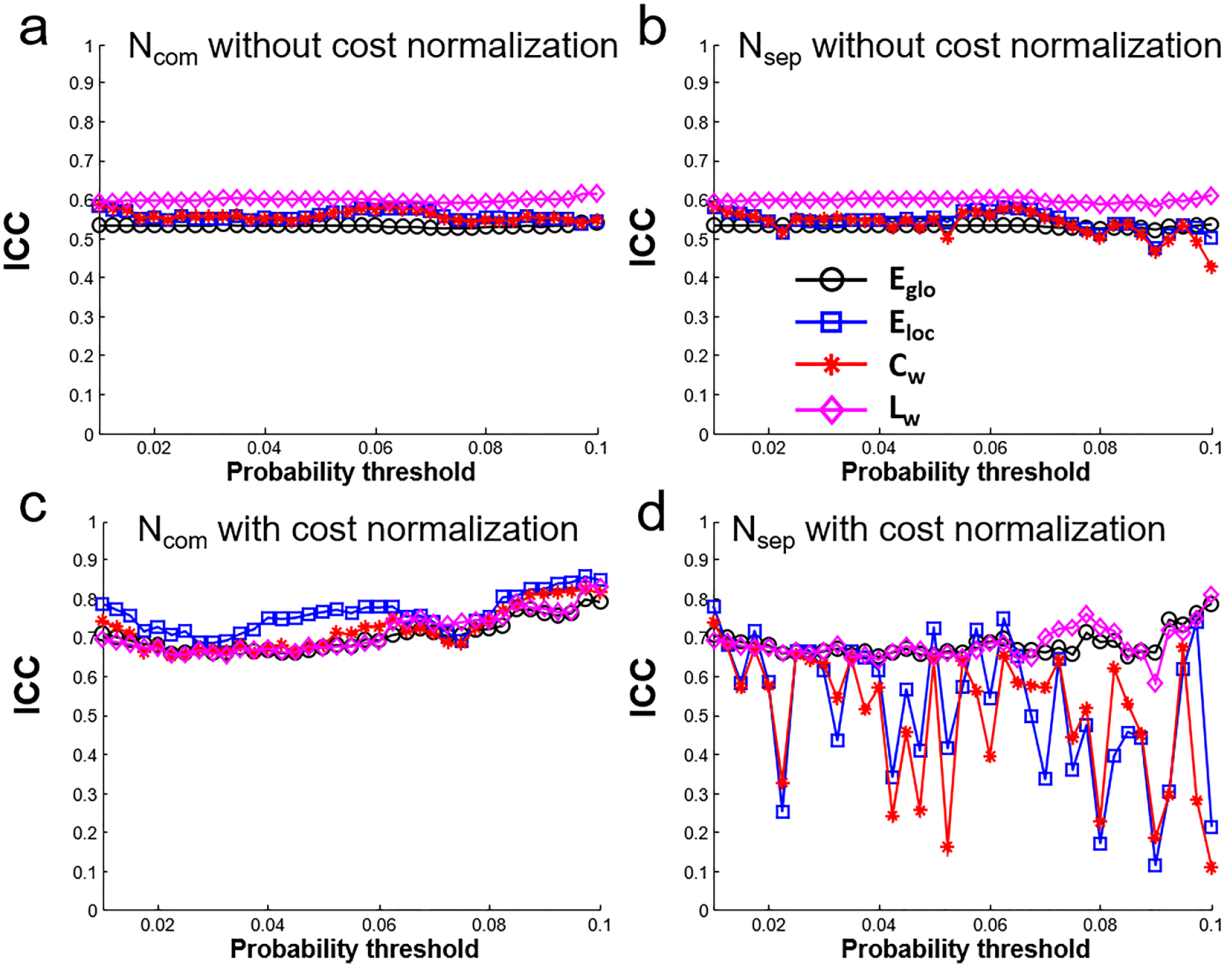
ICC of network metrics (E_glo_, E_loc_, C_w_, L_w_) at 37 connectivity thresholds estimated (**a**,**b**) without cost normalization and (**c**,**d**) with cost normalization. Note The ICC is at level of 0.5 to 0.6 for network metrics without cost normalization and the ICC is at 0.67 to 0.85 for network metrics with cost normalization. However, dramatic variations of ICC are found for E_loc_, C_w_ estimated with cost normalization when sparsity is not the same (N1_sep_ and N2_sep_).

The network metrics (E_glob_, E_loc_, C_p_, L_p_) estimated with and without cost normalization are shown in Fig. 5. Quantitative values are different for the network metrics estimated with and without cost normalization. But the difference among N1_com_, N2_com_, N1_sep_, N2_sep_ for each network metric is small relative to the difference caused by connectivity thresholds by visual inspection. The CV_bs_ and CV_ws_ of network metrics are shown in Fig. 6. When the sparsity is the same (N1_com_ and N2_com_), the CV_bs-com_ range from 6% to 11% and the CV_ws-com_ range from 4% to 7% for network metrics without normalization (Fig. 6a). For network metrics with cost normalization, the CV_bs-com_ are in the range of 1% to 3.5% and the CV_ws-com_ are in the range of 0.4% to 1.8% (Fig. 6b). In general, both the CV_ws-com_ and CV_bs-com_ decreases smoothly as increasing connectivity thresholds (lower sparsity) and the CV_bs-com_ are larger than the CV_ws-com_. When the sparsity is not the same (N1_sep_ and N2_sep_), there is less than 0.1% difference between CV_bs-sep_ and CV_bs-com_ for all connectivity thresholds and for all network metrics estimated with or without cost normalization. Large variation is found between CV_ws-sep_ to CV_ws-com_ in C_w_ and E_loc_ with cost normalization at several connectivity thresholds (up to 3%) (Fig. 6b). The ICC of network metrics are shown in Fig. 7. The ICC of network metrics without cost normalization from N_sep_ and N_com_ are consistent at the range of 0.5 to 0.6 for all connectivity thresholds (Fig. 7a,b). The ICC of network metrics with cost normalization are at range of 0.67 to 0.85 for N_com_ (Fig. 7c). However, the ICC vary in the range of 0.1 to 0.8 through the connectivity thresholds in E_loc_ and C_w_ with cost normalization for N_sep_ (Fig. 7d).

The integrated network metrics are summarized in Table 1. For the network metrics without cost normalization, the CV_bs_ are 6% to 8% and the CV_ws_ are 4% to 5.5%. The ICCs are in the level of fair to good (0.53 to 0.60) for N_sep_ and N_com_. There is only minor difference of CV_ws_ between N_sep_ and N_com_ found on C_w_ (0.13%) and E_loc_ (0.07%). The network metrics with cost normalization show lower CV_bs_ (0.9% to 1.64%) and CV_ws_ (0.7% to 1.05%) compared to those without cost normalization. The ICCs increase but are still in the fair to good level (0.69 to 0.76). A considerable difference of CV_ws_ between N_sep_ and N_com_ is found in C_w_ (0.3%) and E_loc_ (0.15%). That lowers the ICC to the level of 0.5 and 0.63 for C_w_ and E_loc_ in N_sep_.

**Table 1:** Integrated network metrics (E_glo_, E_loc_, C_w_, L_w_) from N1_com_, N2_com_, N1_sep_, N2_sep_ and the associate CV_bs_, CV_ws_, ICC.

without cost normalization		N1	N2	CV_bs_ (%)	CV_ws_ (%)	ICC
com	E_glo_	2.08 ± 0.13	2.07 ± 0.13	6.06	4.12	0.53
	E_loc_	3.28 ± 0.25	3.27 ± 0.25	7.54	5.00	0.55
	C_w_	2.00 ± 0.16	1.99 ± 0.16	7.92	5.26	0.55
	L_w_	884 ± 55.9	885.3 ± 58.2	6.45	4.06	0.60
sep	E_glo_	2.08 ± 0.13	2.07 ± 0.13	6.07	4.12	0.53
	E_loc_	3.29 ± 0.25	3.27 ± 0.25	7.56	5.07	0.54
	C_w_	2.01 ± 0.16	1.99 ± 0.16	7.93	5.39	0.54
	L_w_	884.8 ± 55.9	884.9 ± 58.2	6.45	4.06	0.60
com	E_glo_	(4.82 ± 0.08) × 10^−2^	(4.83 ± 0.07) × 10^−2^	1.49	0.82	0.69
	E_loc_	(7.75 ± 0.07) × 10^−2^	(7.75 ± 0.07) × 10^−2^	0.90	0.44	0.76
	C_w_	(4.73 ± 0.06) × 10^−2^	(4.72 ± 0.06) × 10^−2^	1.27	0.70	0.69
	L_w_	38159 ± 656	38076 ± 593	1.64	0.89	0.70
sep	E_glo_	(4.82 ± 0.08) × 10^−2^	(4.83 ± 0.07) × 10^−2^	1.49	0.83	0.68
	E_loc_	(7.78 ± 0.07) × 10^−2^	(7.74 ± 0.07) × 10^−2^	0.91	0.59	0.63
	C_w_	(4.76 ± 0.06) × 10^−2^	(4.71 ± 0.06) × 10^−2^	1.28	1.05	0.50
	L_w_	38152 ± 659	38052 ± 590	1.64	0.92	0.69

## Discussion

In this study, the short-term reproducibility of structural connectivity matrix among 78 cortical regions estimated using probabilistic tractography was reported based on the connectivity. The edges with higher connectivity have less between-subject variations (lower CV_bs_) and better reproducibility (lower CV_ws_) (Figs 2 and 3). The connectivity threshold of 0.01 excluded around 80% of the edges and these edges show poor to moderate reproducibility (CV_ws_ = 73.2 ± 37.7%; CV_bs_ = 119.3 ± 44.0%; ICC = 0.62 ± 0.19) compared to the other 20% edges (connectivity >0.01) showing moderate to good reproducibility (CV_ws_ < 45%; CV_bs_ < 90%; ICC = 0.75 ± 0.12). A connectivity threshold at 0.01 can be considered as good choice to enroll edges for network analysis. The short-term reproducibility of network metrics (C_w_, L_w_, E_loc_, E_glo_) show smooth change for the connectivity thresholds between 0.01 to 0.1 at the same sparsity (Fig. 6). We found that the network metrics estimated without cost normalization have moderate reproducibility (CV_ws_ < 5.5%, CV_ws_ < 8.0%, ICC = 0.5~0.6) (Table 1) and is less sensitive to the difference in the sparsity. The procedure of cost normalization can improve the reproducibility (ICC = 0.69 to 0.76) in associate with reduced within and between subject variations (CV_ws_ < 1.1% and CV_bs_ < 1.7%). The presence of 1% difference in the sparsity (Fig. 4b,c) can cause additional within subject variations on the estimation of C_w_ and E_loc_ with cost normalization (Fig. 6b), which leads to lower ICC (Fig. 7d). The ICC is still in the moderate level when the networks metrics are estimated over various connectivity thresholds (Table 1).

### Short-term reproducibility of connectivity matrix

For connectivity matrixes, the edges with the higher connectivity mostly coming from the links with shorter tracking distance have better reproducibility (lower CV_ws_) and less inter-subject variation (lower CV_bs_). One reason is that tracking for longer distance is more likely to be interrupted by the successive tracking process, which yield lower connectivity and larger variations. The findings are in agreement with previous reports^27^. The outcome also indicates that tracking using probabilistic model between cortical regions are still subject to the intricate fiber pathways in the brain especially when the seeding points are mostly belonging to gray matter where the fiber has uncertain direction. Connectivity thresholds is therefore necessary to exclude the edges with low connectivity, which can possibly lead to unreliable parameters calculated based on the connectivity matrix. The information of the reproducibility based on the level of connectivity provided in this study can be useful in setting the connectivity thresholds to exclude edges depending on the sensitivity needed for successive analysis. For example, we found that the variations of CV_ws_ and CV_ws_ drop at the connectivity threshold of 0.1. For the study directly using inter-region connectivity for statistical analysis, we suggest to use the connectivity threshold at 0.1, where CV_ws_ < 15% and CV_bs_ < 32% can be reached for 173 edges (5.76%).

### Short-term reproducibility of network metrics

For network metrics estimated at the same sparsity (N1_com_ and N2_com_), both CV_ws-com_ and CV_bs-com_ decrease smoothly in comparable trend as increasing connectivity thresholds (Fig. 6), so the ICC are at same level for all connectivity thresholds and for all network metrics (Fig. 7a,c). The finding implies that the choice of connectivity thresholds in the range of 0.01 to 0.1 may not be crucial for the short-term reproducibility of the estimated network metrics at the same sparsity. Around 1% difference in the sparsity is found between N1_sep_ and N2_sep_ (Fig. 4b,c). The difference in sparsity here is from the difference in connectivity for edges in the shorter-term repetition. Our findings show that the difference in the sparsity leads to relatively larger variation on the CV_ws-sep_ and ICC on E_loc_ and C_w_ with cost normalization (Figs 6b and 7d). Even though, the CV_ws-sep_ of the integrated E_loc_ and C_w_ are still less than CV_bs-sep_ and give moderate reproducibility (ICC > 0.5). Therefore, integration over various connectivity thresholds for the estimation of E_loc_ and C_w_ with cost normalization is suggested when the connectivity thresholds are applied without maintaining sparsity.

### Comparison with the previous literature

Among all studies reporting the reproducibility of network metrics, we compared our results to those using probabilistic tractography and weighted network. In Bucchanan *et al*.^26^, they have reported the test-retest reliability of network metrics using three kinds of definition in network weighting. The CV_ws_ is 3.6% to 4.8% for L_w_ and 5.3% to 7.2% for C_w_. The CV_bs_ is 4.5% to 7.1% for L_w_ and is 8.5% to 9.6% for C_w_. The ICC of L_w_ and C_w_ are in the moderate-to-good range (0.59 ~ 0.76). Andreotti *et al*.^23^ have reported the CV_ws_ for network metrics calculated by averaging over a range of density thresholds. The definition of network weighting is similar to our study but the range of sparsity differs from our study. According to their results, the CV_ws_ of L_w_, E_loc_, E_glo_ is at the level of 4% and CV_ws_ of C_w_ is around 8%. The ICC is the moderate-to-good range (0.66 ~ 0.89). Despite there is difference in network processing procedures between our study and these two previous reports, the CV_ws_ of network metrics without cost normalization are close to their reports (Table 1). Both of these two previous reports found higher CV_ws_ for C_w_ than other network metrics. Same trend is found in our study. The ICC reported in Andreotti *et al*.^23^, Bucchanan *et al*.^26^ and our study are in different levels where our study show lowest ICC among the three studies. The possible explanation is the difference in the subject groups. This leads to difference in the between subject variations. One should notice that the between subject variation (CV_bs_) in the subject sample of this study is expectedly much lower than the general healthy groups because the study population is very homogeneous (healthy young subjects at comparable age and educational level)^23,25,26^. The smaller within and between subject variation helps in inferring the methodological difference in the construction of structural network and in the estimation of network metrics.

### Limitation

In this study, probabilistic tractography is preferred based on previous reports^26,27,31^. Even our analyses were not able to directly compare the deterministic and probabilistic tractorgraphy, there are still other works reporting acceptable repeatability for network metrics from deterministic tractography^28,32,33^. Here, the weighting of the network is defined according the most commonly used weighting scheme in the literatures^21,24,33^. However, the weighting may include effect of both relevant and irrelevant diffusion characteristics of WM tracks. It is therefore difficult to correctly interpret the weight of network. Question on how the definition of the weighting influence the reliability and which weight is more suitable to represent the connectivity are not addressed in this study. The test-retest repeatability of network metrics was investigated in a relative short time interval (~ 30 min.). The subjects stay in the scanner for consecutive measurement makes the CV_ws_ smaller than other studies where repeated scans were made in different days^24,27^. it is also important to understand the long-term or cross site repeatability^24,27^.

In conclusion, we suggest to apply the connectivity thresholds to exclude spurious connections for network analysis. When the sparsity is the same, a connectivity threshold over 0.01 can serve as an acceptable choice without significant effect on the short-term reproducibility of network metrics. When the sparsity is not the same for subject group, the procedure of integration over various connectivity thresholds can be considered to give reliable estimation of network metrics.

## Methods

### Data acquisition

Experiments were conducted in 30 healthy volunteers (15 females/15 males; mean age ± standard deviation: 22.03 ± 1.82 years; age range: 20–26 years). Before being included in the study, all participants gave their informed consent to the protocol, which was approved by the Research Ethic Committee of National Chengchi University. All experiments were performed in accordance with the approved guidelines. Data were collected on a 3T MR system (Skyra, SIEMENS Medical Solutions, Erlangen, Germany) with a 32-channel head coil array. We acquired a high-resolution 3D MPRAGE (Magnetization Prepared Rapid Acquisition Gradient Echo) anatomical scan (TR/TE/flip angle: 2530 ms/3.03 ms/7 degrees; FOV: 224 × 256 × 192 mm^3^; voxel size: 1 × 1 × 1 mm^3^). DTI datasets were obtained using single shot spin echo EPI sequence. We used 30 diffusion directions with b-value 1000 s/mm^2^ and 5 additional images with b-value = 0 s/mm^2^. Experiment parameters were TR = 8800 ms, TE = 90 ms, FOV = 256 × 256 mm^2^, MAT = 128 × 128, slice thickness = 2 mm, slice = 61, NEX = 4, acceleration factor = 3. The total acquisition time were 30 minutes. DTI protocols were repeated twice on each subject for the assessment of short-term test-retest reproducibility (N1 and N2). The subjects were asked to stay in the scanner and rest for 30 minutes between N1 and N2.

### DTI analysis and tractography

Before DTI analysis, 78 cortical regions (39 for each hemisphere) were extracted using the automated anatomical labeling (AAL) template in standard MNI space^34^. Labels and names of cortical regions can be found in Supplementary Table S1. Each cortical region represents a network node. These AAL masks were then transformed to DTI native space for each individual using following procedures. First, T1 images were coregistered to non-diffusion weighting image of DTI data sets. The coregistered T1 image was transformed to standard MNI template using nonlinear transformation. The transformation matrix was then applied to warp the defined AAL mask to DTI native space for each subject. The procedures were carried out using FLIRT and FNIRT tool (FSL, version 4.1; http://www.fmrib.ox.ac.uk/fsl). The AAL masks in each subject were further refined by removing WM voxels that are not neighbor to GM voxels^15^.

For the analysis of DTI data sets, the procedures were performed using the FMRIB Software Library (FSL, version 4.1; FMRIB’s Diffusion Toolbox [FDT]^5^; Oxford Centre for Functional MRI of the Brain [FMRIB], UK; http://www.fmrib.ox.ac.uk/fsl) as described in previous studies^15,21,26^. DICOM images were converted to Neuroimaging Informatics Technology Initiative (NIFTI) format using the MRICron tool. Images were visually checked for observable artifacts, and no volume was discarded. Eddy current correction was applied with the eddy_correct tool, using the default settings. In summary, the first non–diffusion-weighted image was set as the target image, into which the remaining images (120 diffusion weighted image and 3 non-diffusion weighted images) were registered using an affine transformation to adjust for distortions caused by eddy currents and head motion. Then bet tool was used for skull stripping and bedpostx tool was used to build up a two-fibre per voxel model for fiber tracking. Probabilistic tractography was applied to estimate the connectivity probability among 78 cortical regions using PROBTRACKS tool. For the seed region. 5000 fibers streamlines grow from each voxel with tracking parameters of 0.5 mm step size, 500 mm maximum trace length, ±80° curvature threshold. This yields 5000 × n streamlines from the seed region where n is the number of voxel in seed region^5^. The connectivity probability between seed region *i* and target region *j* was then calculated as the ratio of number of fibers passing through target region *j* to the total number of fibers from seed region *i*. The probability from *i* to j is not equal to that from *j* to *i* but they are correlated (all Pearson >0.92, p < 10^−15^). We then calculated the unidirectional connectivity probability between region i to region j (P_ij_) as the average of these two probabilities^15,21^. For each subject a 78 × 78 symmetric connectivity matrix was estimated.

### Construction of weighted network

The connectivity matrix has non-zero probabilities for all 3003 edges. Thresholds on connectivity were used to remove spurious connections that have small connectivity probabilities. Two cortical regions were considered unconnected and set to zero in the connectivity matrix, if the mean connectivity probability across subjects plus two times of standard deviation is less than the connectivity threshold. The sparsity defined as the number of non-zero edges divided by the total number of edges in the diffusion connectivity matrix can be calculated. To ensure same sparsity for N1 and N2, connectivity thresholds were applied on N1 and N2 group together denoted as N1_com_ and N2_com_:

1 $${\rm{average}}({P}_{ij}^{Total})+2std({P}_{ij}^{Total}) < threshold.$$

where ${P}_{ij}^{Total}$ is the connectivity probability from N1 and N2 (60 DTI scans from 30 subjects). In this way, all subjects have the same number of edges and the edges are located at the same position of the connectivity matrix. Further, we applied the connectivity thresholds on N1 and N2 separately, denote as N1_sep_ and N2_sep_:

2 $${\rm{average}}({P}_{ij}^{N1})+2\ast std({P}_{ij}^{N1}) < threshold$$

3 $${\rm{average}}({P}_{ij}^{N2})+2\ast std({P}_{ij}^{N2}) < threshold$$

where ${P}_{ij}^{N1}$ and ${P}_{ij}^{N2}$ is the connectivity from N1 (30 DTI scans from 30 subjects) and N2 (30 DTI scans from 30 subjects) respectively. The resultant connectivity matrixes may have different sparsity for the repeated scans under the same connectivity threshold. This is to examine the potential variation of the network metrics when sparsity cannot be adjusted at same level by the connectivity thresholds. A series of connectivity thresholds at the range of 0.01 to 0.1 with 0.0025 steps were chosen based on Gong *et al*.^15^. The range of sparsity in this study is 8% to 32%, which is similar to previous studies^15,35^. To construct the weighted network from connectivity matrix, the weights of each edge is computed as w_ij_ = P_ij_. For each subject, 37 weighted networks were constructed corresponding to 37 connectivity thresholds.

### Network measures

For each network, four network metrics were calculated. They are network cluster coefficient (C_w_), characteristic path length (L_w_), global efficiency (E_glob_) and local efficiency (E_loc_)^36,37^. The definition of these network metrics is given in the supplementary methods. Because these network metrics were computed for a range of sparsity under a series of connectivity thresholds, the summary network metrics were integrals of each metrics over the range of the sparsity. The network metrics were additionally calculated using the weights scaled by the sum of all weights for all edges to control each subject’s cost at same level, known as cost normalization^15,21^. All network analysis was performed in Matlab (The MathWorks, Natick, USA) using Brain Connectivity Toolbox^9^.

### Quantification of reproducibility

The similarity was calculated by the Pearson’s correlation coefficients of connectivity from structural connectivity matrixes. Within subject similarity was computed by averaging 30 correlation coefficients of connectivity calculated between 2 repeated measurements. Between subject similarity was computed by averaging correlation coefficients of connectivity calculated among 30 subjects. The CV and ICC were used as indices of reproducibility^23,25^. The CV is defined as the standard deviation divided by the overall measurement mean. For CV_ws_, the standard deviation was calculated between 2 repeated measurements (N1 and N2) for each subject. The CV_ws_ was given by mean of within subject standard deviation divided by the overall mean. For CV_bs_, the standard deviation was calculated among 30 subjects in N1 and N2, respectively. The CV_bs_ was given by mean of between subject standard deviation divided by the overall mean. The ICC was computed using two-way mixed single measures using the absolute agreement within the repeated measurement. The ICC was computed using MATLAB toolbox created by Arash Salarian (www.mathworks.com/matlabcentral/fileexchange/22099). The ICC values are classified as: poor reproducibility (<0.5), moderate reproducibility (0.5–0.75), good reproducibility (0.75–0.9), excellent reproducibility (>0.9)^23,38,39^. To summarize the structural connectivity matrix, edges were separated into 31 groups according to the connectivity probability from 0 to 0.3 in 0.01 step. The indexes of the reproducibility were calculated for each group.

## Electronic supplementary material

Supplementary Information
