# Supplementary material for: Reproducibility of structural brain connectivity and network metrics using probabilistic diffusion tractography
Source: Sci Rep. 2018 Aug 1;8:11562. doi: 10.1038/s41598-018-29943-0 (PMC6070542; doi:10.1038/s41598-018-29943-0)
Supplement: Supplementary file 1 — Supplementary Information [file 41598_2018_29943_MOESM1_ESM.docx]

Reproducibility of structural brain connectivity and network metrics using probabilistic diffusion tractography

Shang-Yueh Tsai ^1,2*^

^1^ Graduate Institute of Applied Physics, ^2^ Research Center for Mind, Brain and Learning, National Chengchi University, Taipei, Taiwan

Correspondence to: Shang-yueh Tsai, Ph.D.

Graduate Institute of Applied Physics,

National Chengchi University

NO.64,Sec.2,ZhiNan Rd.,Wenshan District, Taipei 11605,Taiwan

(Tel) +886-2-29393091#62970

(Fax) +886-2-29387769

E-mail: [sytsai@nccu.edu.tw](mailto:sytsai@nccu.edu.tw)

**Supplementary Table S1**. The order of cortical regions in the diffusion connectivity matrix.

| Cortical regions | **Abbreviations** |
| --- | --- |
| Gyrus Rectus | **REC** |
| Olfactory Cortex | **OLF** |
| Supeiror frontal gyrus, orbital part | **ORBsup** |
| Superior frontal gyrus, medial orbital | **ORBsupmed** |
| Middle frontal gyrus orbital part | **ORBmid** |
| Inferior frontal gyrus, orbital part | **ORBinf** |
| Superior frontal gyrus, dorsolateral | **SFGdor** |
| Middle frontal gyrus | **MFG** |
| Inferior frontal gyrus, opercular part | **IFGoperc** |
| Inferior frontal gyrus, triangular part | **IFGtriang** |
| Superior frontal gyrus, medial | **SFGmed** |
| Supplementary motor area | **SMA** |
| Paracentral lobule | **PCL** |
| Precentral gyrus | **PreCG** |
| Rolandic operculum | **ROL** |
| Postcentral gyrus | **PoCG** |
| Superior parietal gyrus | **SPG** |
| Inferior parietal, but supramarginal and angular gyri | **IPL** |
| Supramarginal gyrus | **SMG** |
| Angular gyrus | **ANG** |
| Precuneus | **PCUN** |
| Superior occipital gyrus | **SOG** |
| Middle occipital gyrus | **MOG** |
| Inferior occipital gyrus | **IOG** |
| Calcarine fissure and surrounding cortex | **CAL** |
| Cuneus | **CUN** |
| Lingual gyrus | **LING** |
| Fusiform gyrus | **FFG** |
| Heschl gyrus | **HES** |
| Superior temporal gyrus | **STG** |
| Middle temporal gyrus | **MTG** |
| Inferior temporal gyrus | **ITG** |
| Temporal pole: superior temporal gyrus | **TPOsup** |
| Temporal pole: middle temporal gyrus | **TPOmid** |
| Parahippocampal gyrus | **PHG** |
| Anterior cingulate and paracingulate gyri | **ACG** |
| Median cingulate and paracingulate gyri | **DCG** |
| Posterior cingulate gyrus | **PCG** |
| Insula | **INS** |

**Supplementary Methods**

For a node, the likelihood that the neighborhoods were connected with each other is defined as cluster coefficient and the average of cluster coefficient for all nodes is the C_w_, which indicates the extent of local connectivity in a network.

$$C_{w}\left( G \right)=\frac{1}{N}\sum_{i\epsilon G} \frac{2}{k_{i}(k_{i}-1)}\sum_{j,k} {(w_{ij}w_{jk}w_{ki})}^{1/3} (1)$$

Where G is the network, $k_{i}$ is the degree of the node.

The shortest path (d_ij_) between each pair of nodes within the network is defined as the shortest length of path for node i and node j, where the lengths of each edge was the reciprocal of the edge weights, 1/w_ij_. The characteristic length of network, L_w_, indicate the ability for information to be propagated in parallel,

$$L_{w}\left( G \right)=\frac{1}{N\left( N-1 \right)}\sum_{i\neq j\epsilon G} d_{ij} (2)$$

Other than typical network parameters for unweighted network, concept of network efficiency was introduced in the weighted network. E_glob_ of the network is the average of all pair wise reciprocal of shortest path (d_ij_) between each pair of nodes within the network, which indicates the efficiency of the parallel information communication in the network.

$$E_{glob}\left( G \right)=\frac{1}{N\left( N-1 \right)}\sum_{i\neq j\epsilon G} \frac{1}{d_{ij}} (3)$$

Global efficiency is calculated for each subgraphs G_i_ which is the small network containing node i and its neighborhoods. The average of global efficiency from all subgroup is E_loc_, which indicates the fault tolerance for the network and shows the efficiency of communication among the first neighbors of a node I when it is removed.

$$E_{loc}\left( G \right)=\frac{1}{N}\sum_{i\epsilon G} E_{glob}\left( G_{i} \right) (4)$$
